# P-814. Utility of PET-CT Scan in *Staphylococcus aureus* Bacteremia

**DOI:** 10.1093/ofid/ofae631.1006

**Published:** 2025-01-29

**Authors:** Christopher Peterson, Mamata Reddy Tokala, Ruth Ndolo, Christopher Stocki, Sydney Stayrook, John-Paul O’Shea, Ekta Bansal, Mariana Gomez de la Espriella, Tasaduq Fazili

**Affiliations:** Virginia Tech Carilion School of Medicine, Roanoke, Virginia; Carilion Healthcare, Fuquay Varina, North Carolina; Carilion Clinic, Roanoke, Virginia; Virginia Tech Carilion School of Medicine, Roanoke, Virginia; Virginia Tech Carilion School of Medicine, Roanoke, Virginia; Virginia Tech Carilion School of Medicine, Roanoke, Virginia; Virginia Tech Carilion School of Medicine, Roanoke, Virginia; Carilion Clinic, Virginia Tech, Salem, Virginia; Virginia Tech Carilion School of Medicine, Roanoke, Virginia

## Abstract

**Background:**

*Staphylococcus aureus* bacteremia is a common yet serious infection with high morbidity and mortality. The timely identification of the primary source of infection and any associated metastatic foci is crucial for guiding appropriate management strategies and improving patient outcomes. Positron emission tomography-computed tomography (PET-CT) scans offer a non-invasive imaging modality that combines the metabolic information provided by PET with the anatomical detail provided by CT. Our investigation aims to show that PET-CT is a useful modality for improving mortality in patients with *S. aureus* bacteremia.

**Table 1:**
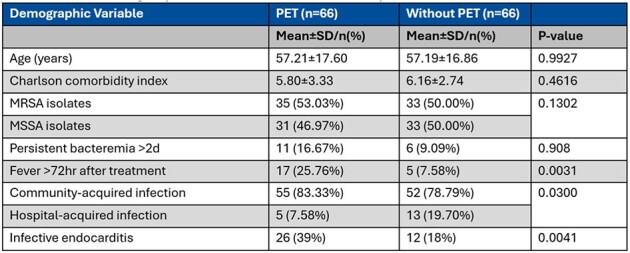
Demographic Variables of Participants

**Methods:**

Patients with *S. aureus* bacteremia who underwent PET-CT at Roanoke Memorial Hospital between November 2020 and November 2022 were identified through electronic health records. These were matched with a control group comprised of patients with *S. aureus* bacteremia who did not undergo PET-CT scans. Logistic regression analysis was employed for statistical comparison between groups.

**Table 2:**
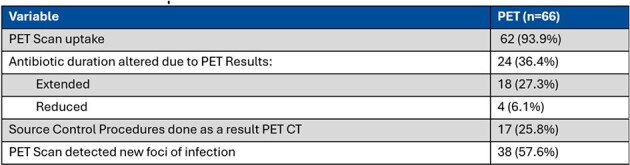
PET Group Results

**Results:**

A total of 132 patients were included in the study, with 66 in both the PET group and the group without PET (Table 1). Patients undergoing PET-CT had significantly lower odds of mortality compared to those without the scan (OR=0.404, 95% CI: 0.167 - 0.980, p=0.0451). In the PET-CT group, PET-CT detected a new foci of infection in 57.6% of cases and a source control procedure was performed in 25.8% of cases as a result of PET-CT findings (Table 2). Subgroup analysis within the PET group showed positive PET scan uptake in the majority of patients (93.94%), with whole-body PET-CT being the most commonly performed imaging protocol. Antibiotic duration was adjusted in 33% of patients based on PET-CT findings. Patients in the PET-CT group received a longer course of antibiotics (36.8 vs 29.3 days)(Table 3).

**Table 3:**
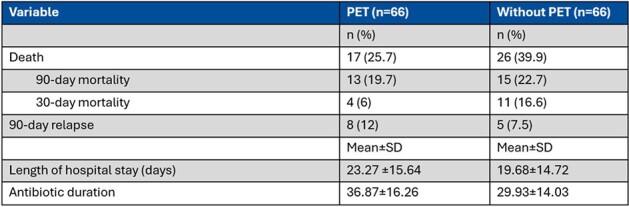
Primary and secondary outcomes

**Conclusion:**

These results underscore the potential clinical utility of PET-CT in guiding therapeutic interventions. Here we observed a lower mortality in patients with *S. aureus* bacteremia who underwent PET-CT imaging, as well as increased performance of source control procedures and longer antibiotic duration. PET-CT scans offer a comprehensive assessment of infection localization assisting clinicians in identifying occult foci of infection.

**Disclosures:**

**All Authors**: No reported disclosures

